# Autophagy inhibition improves the cytotoxic effects of receptor tyrosine kinase inhibitors

**DOI:** 10.1186/s12935-018-0557-4

**Published:** 2018-04-24

**Authors:** Sanja Aveic, Marcella Pantile, Pierfrancesco Polo, Viktoryia Sidarovich, Marilena De Mariano, Alessandro Quattrone, Luca Longo, Gian Paolo Tonini

**Affiliations:** 1 Neuroblastoma Laboratory, Fondazione Istituto di Ricerca Pediatrica Città della Speranza, Padua, Italy; 2 University of Padua, Padua, Italy; 3 Centre for Integrative Biology, University of Trento, Trento, Italy; 4 UOC Bioterapie, Ospedale Policlinico San Martino, Genoa, Italy

**Keywords:** Neuroblastoma, RTK inhibitors, Autophagy, Drug combination

## Abstract

**Background:**

A growing field of evidence suggests the involvement of oncogenic receptor tyrosine kinases (RTKs) in cell transformation. Deregulated activity of RTKs in tumors can determine disease progression and therapeutic responses in several types of cancer, including neuroblastoma (NB). Therefore, RTKs targeting is a worthwhile challenge for the oncologists. Nevertheless, acquired resistance to RTK inhibitors (RTKi) remains a serious problem. Autophagy activation is among the possible obstacles for good efficacy of the therapy with RTKi.

**Methods:**

Under different treatment conditions we measured autophagic flux using immunoblot and immunofluorescence assays. Death induction was validated by trypan blue exclusion assay and FACS analysis (calcein-AM/propidium iodide). The NB cell lines SH-SY5Y and Kelly were used for the in vitro study.

**Results:**

In order to define whether autophagy might be a limiting factor for the efficacy of RTKi in NB cells, we firstly checked its activation following the treatment with several RTKi. Next, we investigated the possibility to increase their therapeutic efficiency by combining RTKi with autophagy blocking agents in vitro. We exploited the effectiveness of three RTKi either alone or in combination with autophagy inhibitors (Chloroquine—CQ and Spautin-1). We demonstrated that autophagy induction was drug-dependent, and that its inhibition increased the anti-tumor activity of a single RTKi unevenly. We observed that the combined use of blocking agents which impair late autophagy events, such as CQ, and RTKi can be more effective with respect to the use of RTKi alone.

**Conclusions:**

In the present report, we assessed the conditions under which autophagy is activated during the use of different RTKi currently in the pre-clinical evaluation for NB. We summarized the achievements of combined RTK/autophagy inhibitors treatment as a promising approach to enhance the efficacy of RTKi in impairing tumor cells viability.

**Electronic supplementary material:**

The online version of this article (10.1186/s12935-018-0557-4) contains supplementary material, which is available to authorized users.

## Background

Autophagy is an evolutionarily conserved cellular mechanism that allows a recycling of the intracellular, metabolic-related, building blocks necessary for regular function of the cells [1]. It occurs through a complex, multi-regulatory process during which cytosol components are swamped by double-layer membrane vacuoles, fused with lysosomes and degraded [2]. In stressful situations this process is used by the cells to recover momentary disturbed homeostasis, keeping the system in standby until homeostatic rheostat is repaired, or triggering apoptosis if stress is prolonged and overwhelming [3]. Malignant cells undergo substantial stress when patients are under chemotherapy, and in this situation tumor cells may use autophagy to eliminate the drug or resist drug cytotoxicity [4]. As a consequence, autophagy may interfere with expected anti-tumor activity of these drugs [5]. Indeed, a number of anti-cancer compounds can induce cyto-protective autophagy [6]. Beside conventional chemotherapics, protective role of autophagy was also described during the use of receptor tyrosine kinase inhibitors (RTKi) [7, 8]. After the first enthusiasm brought by the introduction of RTKi in cancer treatment which decreased tumor mass, and gave hope for a complete eradication of cancer cells, it emerged that many, if not all, patients developed resistance to the RTKi treatment [9]. The major part of resistance to the first generation of RTKi was acquired after mutations, amplifications or phenotypic changes (such as epithelial-to-mesenchymal transition) that occurred within some populations of tumor cells. However, today there is no precise definition of how the therapy with RTKi needs to be modified once the resistance is confirmed. At the molecular level, the use of RTKi generally leads to the activation of the compensatory mechanisms within the same cell that tend to overcome toxic inputs. Hence, the most adopted strategy in these circumstances is to add another RTKi in order to suppress either of the stimulated pathways. Recently, we have identified several different RTKi as plausibly efficient for neuroblastoma (NB) treatment [10, 11]. NB is a neoplasm of neuroendocrine origin that occurs more commonly in pre-scholar children. This pediatric tumor counts for a high rate of death events if diagnosed in children over 18 months of age or with a late diagnosis of stage 4 metastatic disease [12]. For this group of patients new treatments are required in order to increase current survival rate which is at the moment below 50% at 5-years from diagnosis [13, 14]. Beside well-known negative prognostic markers, such as MYCN, Aurora A, ALK, TrkB and Survivin, novel mutations, predisposing polymorphisms, and other genetic aberrations in RTKs have been described in NB as well, raising these receptors as candidate therapeutic targets in this cancer [15, 16]. Hence, the employment of RTKi that are already in use as the therapy option for solid tumors in adult patients might prove useful also for NB treatment, if the specific target appears to be involved in disease onset. However, the administration of RTKi leads almost inevitably to resistance phenomena that overcome the on-target effects of these inhibitors. Autophagy is one of the mechanism that NB cells may activate to resist the cytotoxic effect of therapy.

Therefore, in the present study we evaluated the induction of autophagy as a consequence of the treatment of NB cells with three different RTKi (Afatinib, Sorafenib and TP-0903), and investigated if the block of autophagy could increase the efficacy of these RTKi.

## Methods

### Cell line and treatments

The maintenance of SH-SY5Y and Kelly cell lines (DSMZ) was done in RPMI medium (Sigma-Aldrich, Italy) supplemented with FBS (fetal bovine serum; 10%), glutamine (1%) and antibiotics (1%; all from Gibco, Life Technologies, Italy). The number of the cells required for the analyses was preliminarily optimized for each experimental setting. The RTKi: Sorafenib, Afatinib and TP-0903 were all purchased from Selleck Chemicals (Germany). Cell count was done on the Countess™ automated cell counter (Invitrogen) using the trypan blue, and the cell viability was measured indirectly by the means of their metabolic activity using tetrazolium compound (MTT). The inhibitory concentration that reduced cell viability by 50% was determined as described in details previously [17].

### RNA isolation and quantitative real time PCR (qPCR)

RNA extraction was performed with TRIzol reagent according to manufacturer’s recommendation (Invitrogen, Life Technologies). Complementary DNA was prepared with SuperScript II following a procedure described by manufacturer (Invitrogen). qPCR was carried out on Applied Biosystems FAST Real-Time PCR system 7900 using SYBR Green master-mix (Applied Biosystems, Forest City, CA). Cycle quantification and relative expression measurement for *BNIP3* mRNA was performed using 2^−∆∆Cq^ method as explained elsewhere [18]. The *GAPDH* expression was used as internal normalizing control. The primer sequences are available upon request.

### Immunostaining and necrosis detection

Autophagosomes were detected by Autophagy Detection Kit (Abcam, Italy). The protocol adapted for immunofluorescence microscopy was applied for the staining of the autophagy vacuoles (green). The cells were grown on 4-wells chamber-slides (150,000 cells/well). Hoechst dye was used for nuclear marking (blue). Images were obtained by Nikon (Vico, Eclipse Ti80, Tokyo) under 60X magnification, using oil immersion objective. Percentage of necrotic cells was determined by calcein-AM/propidium iodide (PI; Sigma-Aldrich) staining using flow cytometry (Becton and Dickinson, Heidelberg, Germany). Cells were incubated for 30 min with calcein-AM (1 mg/ml) and PI (10 mg/ml). Minimum 20,000 events were acquired for each sample. The percentage of necrotic, PI positive, cells was distinguished from the total cell population.

### Protein extraction and immunoblot

Cells were trypsinized, washed well in PBS and pelleted before adding cold lysis buffer (Biosource International; Camarillo, CA) containing 1× protease and phosphatase inhibitors (Sigma-Aldrich). Quantification was done using BCA protein quantification kit (Thermo Fisher, Italy) as described by the manufacturer. Incubation for 30 min with reaction reagent was done at 37 °C to stimulate colorimetric reaction, and absorbance was then measured on VICTOR plate reader (486 nm). A total of 20 µg of proteins were loaded on 4–20% gradient gel and SDS-PAGE (Bio-Rad, Italy) was done as described in details elsewhere [17]. Primary antibodies used in the study were anti-: MCL1, PARP, total and phospho ERK, phospho AKT, phospho PI3K, phospho mTOR, BCL2, BCL-XL Caspase-3 (Cell Signaling), LC3, GAPDH, BECLIN 1, ATG5 (Novus Biologicals, Littleton, CO), p62/SQSTM1 (Abnova, Taipei City, Taiwan), PCNA (SCBT, Dallas, TX) using dilutions suggested by the manufacturer. Where necessary, a densitometry was done (ImageJ software from the National Institutes of Health; Bethesda, MD was used), using the expression of GAPDH for data normalization.

### Clonogenic assay

Cells were seeded with Methocult H4100, previously prepared adding 40 ml of Methocult in 60 ml of RPMI medium, in 12-well plates at a concentration of 2000 cells/well. Cells were incubated with either Afatinib (8 µM) or Sorafenib (14 µM), as well as with CQ (25 µM) and SP1 (10 µM) alone, and with each combination of RTKi and autophagy inhibitors. DMSO was used as a control. Cells were grown for 2 weeks followed by 4 h long MTT staining and colonies count. Colony numbers were represented as the mean ± SD of three replicates.

### Statistical analysis

Data were obtained from at least three independent experiments and presented as mean ± SD. The results acquired for the RTKi-treated samples were compared to the control, DMSO treated samples. Statistical significance was evaluated by one-way ANOVA with post hoc Dunnett’s or Tukey’s multiple comparison test (GraphPad version 4.0). The p values < 0.05 (95% confidential interval) were considered statistically significant and results were presented as *p < 0.05; **p < 0.01 and ***p < 0.001.

## Results

### RTKi exert different effects on SH-SY5Y cells

Recently we concluded a high-throughput screening of an anti-tumor drug library that consists of 349 small molecules in order to select the compounds that are efficient against NB [11]. We came upon four different RTKi that were considered for further pre-clinical evaluations. Two of them that resulted to be less effective in impairing NB cell survival, namely Afatinib and Sorafenib, were chosen for this study. We aimed to evaluate whether less effective cytotoxic effects observed for Afatinib and Sorafenib were caused by autophagy induction. Additionally, we included another RTKi, named TP-0903, for which we also showed to have excellent anti-tumor characteristics [10]. In order to evaluate if these compounds can activate cyto-protective autophagy in NB, we employed SH-SY5Y cell line as an in vitro model, known to activate autophagy upon treatment with ALK inhibitors such as Entrectinib and Crizotinib [17]. For each of the selected RTKi at first we defined the toxic concentration range, and calculated the half maximal inhibitory concentration (IC_50_; Table 1). While Afatinib and Sorafenib were active at the µM range, TP-0903 worked in sub-µM concentrations. Thereafter, the IC_50_, and two sub-toxic concentrations, were selected to study a potential role of autophagy in protecting NB cells from the treatment with RTKi.

**Table 1:** Inhibitory concentration 50 (IC_50_)

Drug	IC_50_ at 24 h (mean ± SD) μM
Afatinib	7.7 ± 0.6
Sorafenib	12.4 ± 3.4
TP-0903	0.130 ± 0.009

The concentration that inhibits cell viability for 50% was determine for each RTKi (Afatinib, Sorafenib and TP-0903). The mean values and standard deviations are indicated for 24 h treatment period

### Level of autophagy activation is RTKi dependent

**Fig. 1:**
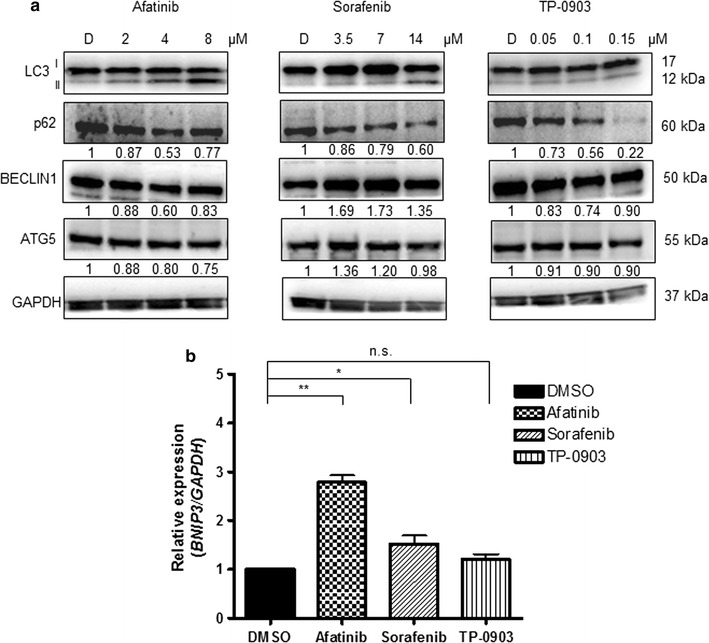
Expression of autophagy regulators upon treatment. **a** Increasing concentrations of Afatinib, Sorafenib and TP-0903 were used for the treatment of SH-SY5Y cells for 24 h (upper numbers indicate the concentrations used in μM; D = DMSO). Levels of main autophagy regulators were evaluated by western blot. GAPDH served as a loading control protein. Numbers under the single protein indicate relative expressions obtained by densitometry measurement, normalized to GAPDH, and compared to the control samples (DMSO = 1). **b** Real-time quantitative PCR (qPCR) was employed for the evaluation of *BNIP3* mRNA level 24 h post-treatment, with the median value from the concentrations defined for each RTKi. *GAPDH* was used as the housekeeping gene. Relative quantification (RQ) was done after normalizing a single Cq to the Cq of *GAPDH,* and ΔΔCq method was then applied to calculate the relative expression in treatments with respect to DMSO treated control. Results are presented as mean ± SD of three independent experiments, and p value marked as *p < 0.05; **p < 0.01; *ns* non-significant

**Fig. 2:**
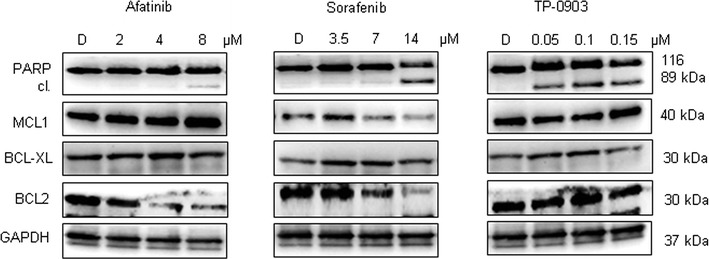
Cytotoxic effects of RTKi at protein level. Increasing concentrations of each RTKi were applied for 24 h. Apoptosis level was measured by means of PARP cleavage, as one of the hallmark of programmed cell death activation The expression of the main anti-apoptotic proteins from BCL-2 family was examined as well. GAPDH served as a loading control protein. *D* DMSO; the numbers indicate the concentrations used in μM

In order to explore whether autophagy activation occurred following the treatment with selected RTKi, we examined the expression of the main autophagy-related proteins: LC3, BECLIN 1, p62/SQSTM1 and ATG5. Afatinib, and at a major extent Sorafenib, led to a clear autophagy activation as confirmed by the conversion of microtubule-associated protein light chain 3 (LC3) form I into LC3-II form, and decreased p62 protein levels. Increase in BECLIN 1 and ATG5 levels were more evident in Sorafenib treated samples. Conversely, TP-0903 affected autophagy at a much lower extent (Fig. 1a). These properties were already detectable at the lowest drug concentrations used in the study, and reached the most evident increase in the LC3-II form after use of the highest drug concentration (IC_50_). The activation of autophagy was additionally confirmed at RNA level where *BNIP3* expression increased significantly only in Sorafenib and Afatinib treated samples (RQ: DMSO = 1; Afatinib = 2.8 ± 0.2; Sorafenib = 1.5 ± 0.3; TP-0903 = 1.2 ± 0.2; Fig. 1b). Eventual cell death activation was then detected measuring poly (ADP-ribose) polymerase-1 (PARP) protein cleavage by western blot. While PARP cleavage was observed only for the cytotoxic doses of Afatinib and Sorafenib, TP-0903 successfully induced cell death already at the sub-IC_50_ concentration determined for SH-SY5Y cells (Fig. 2). Additional evaluation of the Caspase-3 cleavage, and the percentage of propidium iodide (PI) positive cells (Additional file 1: Figure S1A, B), suggested that both types of cell death, apoptosis and necrosis, were activated during the treatment with RTKi. Apoptosis was preferentially activated by Afatinib and Sorafenib, whereas TP-0903 induced both cell death types, apoptosis and necrosis. In addition, the RTKi, especially Sorafenib, affected the cell proliferation as confirmed by reduced expression of the proliferating cell nuclear antigen (PCNA; Additional file 1: Figure S1C). Together, these results implied that either increased cell death or the impairment of cell proliferation in parallel with cell death induction, were a cause of the reduced cell number detected by MTT. The observed changes in cell survival rate were followed by the variations in the expression of the MCL1 and BCL2, the two important anti-apoptotic proteins from BCL-2 family that can influence autophagy induction [19]. Both, MCL1 and BCL2 were significantly down-regulated upon Sorafenib treatment confirming the results already reported in the literature [20]. Afatinib caused a decrease in BCL2 level only, while BCL-XL expression did not change significantly upon any of these treatments. Interestingly, as a consequence of dose dependent reduction of BCL2 level, the increase in BECLIN 1/BCL2 ratio occurred in Afatinib treated samples (DMSO = 1; Af 2 µM = 1.4; Af 4 µM = 1.6; Af 8 µM = 2.8). Hence free BECLIN 1, which is preferentially sequestered by BCL2 [21], was then sufficient to promote autophagy without leading to additional production of BECLIN 1 protein in Afatinib treated NB cells. No significant changes in the expression of any of the BCL-2 family member were observed in TP-0903 treated NB cells. These results confirmed that both, Afatinib and Sorafenib could activate autophagy in SH-SY5Y cells (at sub-IC_50_ doses), and that this activation preceded the induction of cell death that was found active along with autophagy (at IC_50_ doses).

### Inhibition of autophagy increases RTKi efficacy

**Fig. 3:**
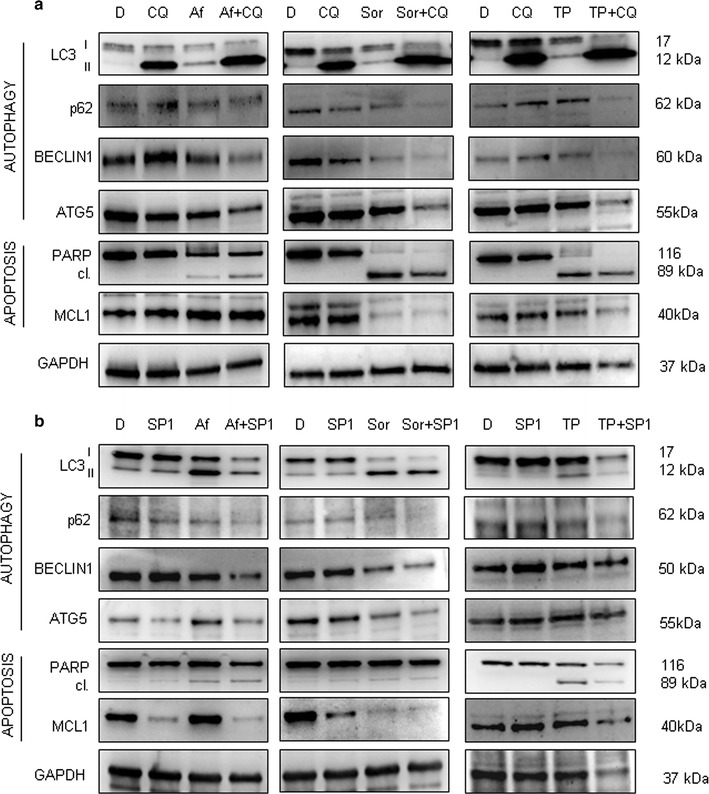
Single versus combined treatment with RTKi and autophagy blocking agents. RTKi were used as a single treatment or in combination with **a** Chloroquine (CQ; 25 μM) or **b** Spautin-1 (SP1; 10 μM). Changes in LC3, BECLIN 1, p62 and ATG5 protein levels were evaluated by western blot. Changes in autophagy and apoptosis activation were validated in parallel. Apoptosis was evaluated by measuring intensity of PARP cleavage. Besides, MCL1 protein expression was checked out in order to determine the capacity of combined treatment to decrease it. GAPDH served as a loading control protein. *D* DMSO, *CQ* Chloroquine, *SP1* Spautin-1, *Af* Afatinib (8 μM), *Sor* Sorafenib (14 μM), *TP* TP-0903 (0.15 μM)

**Fig. 4:**
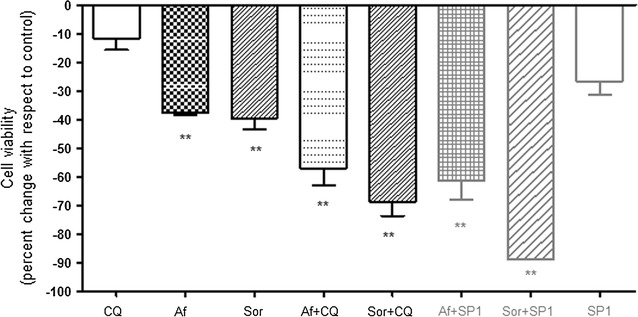
Cell viability assessment upon single and combined treatments. Trypan-blue exclusion assay was applied for the evaluation of the cell viability 24 h post-treatment with single RTKi or in combination with CQ and SP1. The percent change measured with respect to DMSO control (DMSO percent change = 0) is shown. Results are presented as mean ± SD out of triplicates, and p value marked as **p < 0.01; *ns* non-significant (Dunnett’s test done using CQ value as a control for the multiple comparison). *CQ* Chloroquine, *SP1* Spautin-1, *Af* Afatinib, *Sor* Sorafenib

**Fig. 5:**
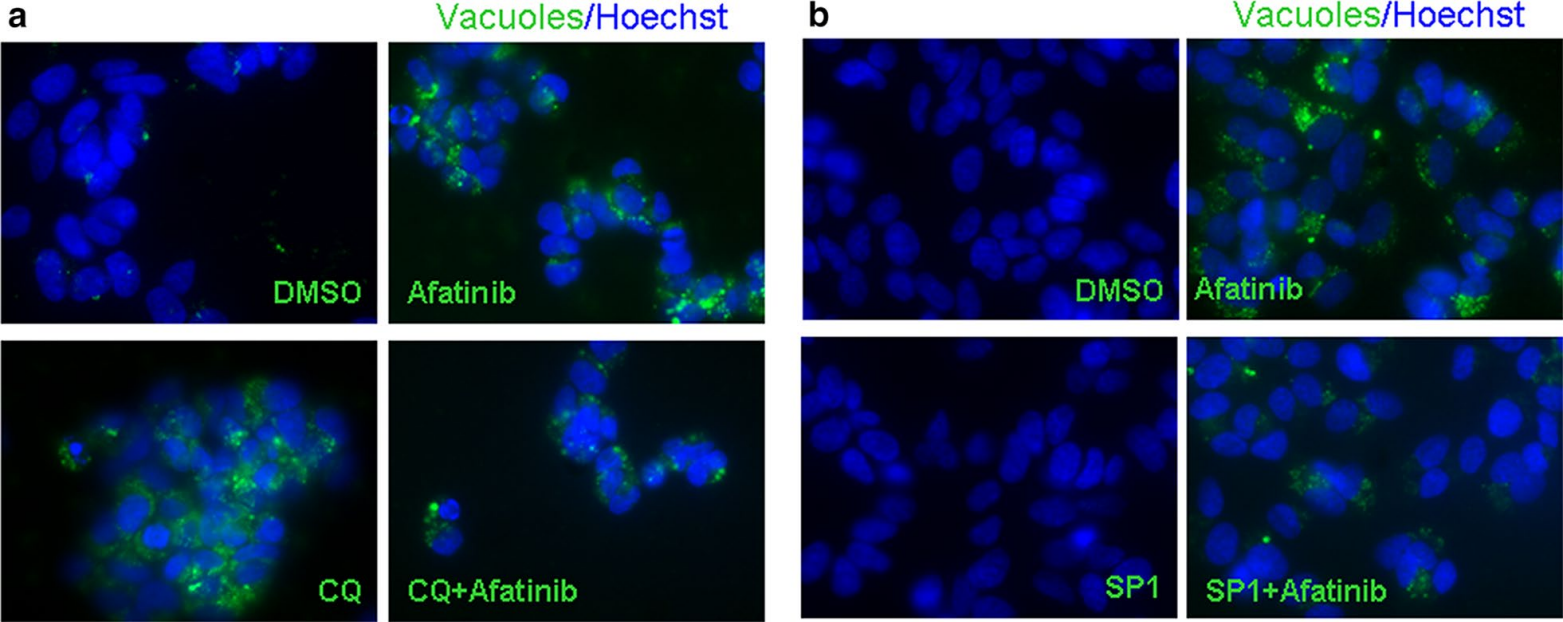
Autophagic vacuoles creation. Autophagy activation upon treatment with Afatinib was additionally validated using immunofluorescence in order to visualize a creation of autophagy vacuoles; ×60 magnification. Green—autophagy vacuoles; Blue—Hoechst, nuclear staining. The combined treatment with Afatinib and **a** CQ or **b** SP1 was used

As observed, the grade of PARP cleavage was concentration dependent for each RTKi, particularly Afatinib and Sorafenib. To confirm that the observed autophagy had a cyto-protective role we used two autophagy inhibitors, Chloroquine (CQ) and Spautin-1 (SP1), prior to treatment with RTKi. Then we evaluated whether apoptosis level changed during combo-treatment with respect to that with a RTKi alone. The changes in the expression of both, autophagy and apoptosis regulatory proteins sustained the efficacy of the combo-treatment (Fig. 3). More precisely, combo-treatment strategy increased the cleavage of PARP protein in Afatinib treated samples, and led to its almost complete cleavage in Sorafenib and TP-0903 treated samples. However, the efficacy of the combined treatment of RTKi with CQ (Fig. 3a) resulted as more powerful than the combination with SP1 (Fig. 3b). In addition, particularly interesting was the decrease in MCL1 level observed upon single and combined treatments with autophagy inhibitor SP1. Cell death induction upon drug addition was sustained by the cell number assessment performed after single or combo-treatment. A cumulative incidence of cell death events in conditions of inhibited autophagy was detected (Fig. 4). Particularly significant were results obtained for Afatinib and Sorafenib (Fig. 4), whereas TP-0903 showed additional, but not statistically significant death induction upon combinatorial conditions (data not shown). Subsequently, we proved previously observed changes in autophagy activation for Afatinib after single or combined treatment using immunofluorescence. We confirmed the creation of autophagosomes in Afatinib treated SH-SY5Y cells, but not in their control (DMSO) counterpart (Fig. 5). Additionally, we observed accumulation of autophagosomes in the presence of CQ (late autophagy block), whereas no autophagosomes were created in SP1 (early autophagy block) treated samples. The combination of Afatinib and CQ did not lead to additional increase in autophagosome creation (Fig. 5a), while combo-treatment with SP1 decreased the number of autophagic vacuoles (Fig. 5b) confirming a different point of action of the two autophagy inhibitors.

### The effects of autophagy inhibition are time dependent

**Fig. 6:**
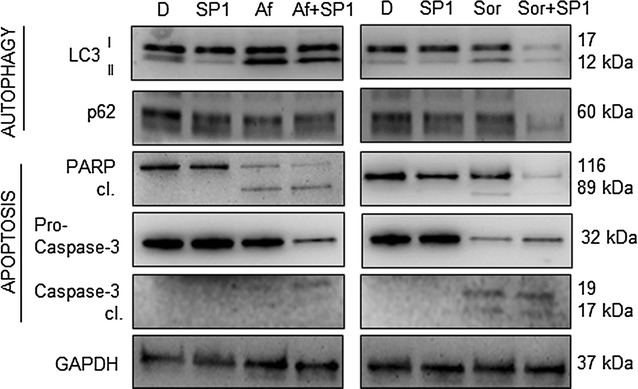
Post-treatment with the SP1 agent. RTKi were used as a single pre-treatment or in combination with Spautin-1 (SP1; 20 μM; added 2 h upon use of RTKi). Changes in LC3, p62, PARP and Caspase-3 protein levels were evaluated by western blot. GAPDH served as a loading control protein. *D* DMSO, *SP1* Spautin-1, *Af* Afatinib (8 μM), *Sor* Sorafenib (14 μM)

To understand whether different concentration or timing of SP1 addition could result in more effective sensitisation of NB cell to the toxic insults of RTKi, we repeated the treatments with the increased dose of SP1 (25 µM), or by modifying the treatment schedule (post-treatment with SP1). While in the former case we did not observe any significant change in the level of cell death activation (data not shown), the latter option gave more promising results. As shown in Fig. 6, the addition of SP1 to the RTKi pre-treated cells provoked more evident changes in the pattern of the main autophagy and apoptosis regulators comparing to the condition in which SP1 was used before the treatment with RTKi. In particular, addition of SP1 to the Sorafenib pre-treated cells, in which autophagy was ongoing, led to a more pronounced autophagy-flux modulation. Also, the level of PARP protein was markedly reduced, and the cleavage of Caspase-3 was more evident for both combinations (Afatinib and SP1, and Sorafenib and SP1) when compared with the results obtained when SP1 was added prior to RTKi treatment (Fig. 3b). These findings indicated that there might be a defined time frame within which the NB cells could be more vulnerable to the specific autophagy inhibitors, and that this therapeutic window should be considered for their use together with RTKi.

### Clonogenic potential of SH-SY5Y cells was abrogated following combo-treatment

Next, we evaluated whether concurrent autophagy block and RTKi application could potentially impact the ability of SH-SY5Y cells to form colonies. For this purpose, NB cells were pre-treated with Sorafenib, Afatinib, CQ or SP1 alone or with pre-determined combinations of these RTKi and autophagy inhibitors, and then plated in semi-solid medium. The number of colonies that formed after 14 days of plating was evaluated for each experimental condition. The results obtained for Afatinib sustained the potential of the combo-treatment to impair clonogenic potential of NB cells, making them less capable to proliferate and form colonies (Additional file 2: Figure S2). The combined use of Sorafenib and autophagy inhibitors was equally effective, abrogating completely the creation of colonies (data not shown).

### Canonical and non-canonical activation of autophagy in SH-SY5Y cells

In order to determine whether selected RTKi induced autophagy via canonical signaling axis only, or also via non-canonical one, we analyzed the expression of PI3K/AKT/mTOR and ERK proteins, respectively [22]. We detected the changes in the PI3K/AKT/mTOR regulatory axis (particularly mTOR expression was impacted upon treatment with selected RTKi), but also in the ERK phosphorylation level (Additional file 3: Figure S3). These findings implied that both modules, PI3K/AKT/mTOR and ERK, were potentially important for autophagy regulation in NB cells.

### Autophagy activation upon RTKi treatment occurs in Kelly cell line as well

**Fig. 7:**
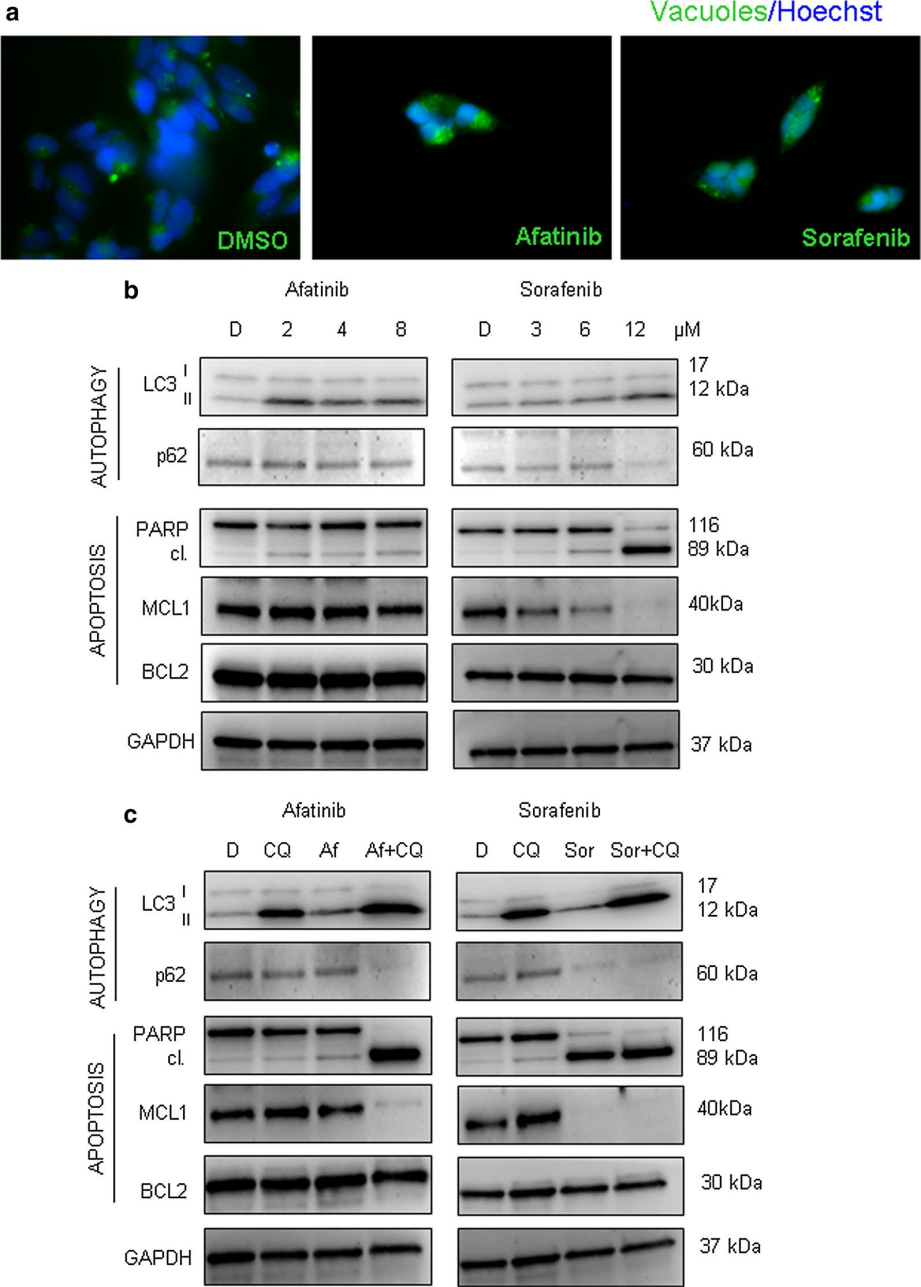
Autophagy activation in Kelly cell line upon treatment with RTKi. **a** Autophagy activation upon treatment with Afatinib and Sorafenib was validated using immunofluorescence in order to visualize a creation of autophagosomes; ×60 magnification. DMSO treatment was used as control. Green—autophagy vacuoles; Blue—Hoechst, nuclear staining. **b** Increasing concentrations of Afatinib and Sorafenib were used for the treatment of Kelly cells for 24 h (numbers indicate the concentrations used in μM). Levels of main autophagy and apoptosis related proteins were evaluated by western blot. GAPDH served as a loading control protein. *D* DMSO. **c** RTKi were used as a single treatment or in combination with CQ (25 μM). Changes in autophagy and apoptosis related proteins were evaluated by western blot. GAPDH served as a loading control protein. *D* DMSO, *CQ* Chloroquine, *Af* Afatinib (8 μM), *Sor* Sorafenib (12 μM)

Finally, in order to assess how RTKi influence autophagy in other NB cell lines, we repeated the treatments using Kelly cells. The two RTKi that significantly affected autophagy in SH-SY5Y, Afatinib and Sorafenib (Additional file 4: Figure S4), induced autophagy also in Kelly cell line. The creation of autophagosomes was observed under microscope (Fig. 7a), and was accompanied by the activation of autophagic flux as confirmed at LC3-II and p62 protein levels (Fig. 7b). More precisely, concentration dependent decrease in the p62 level and appearance of LC3-II were observed. Apoptosis induction was then confirmed by PARP protein cleavage, while the changes in the BCL-2 family proteins occurred only for MCL1 after the treatment with Sorafenib. Combination of RTKi and CQ was particularly successful in Kelly cell line. The autophagy block together with RTKi addition provoked an evident increase in cell death occurrence, which not only induced additional cleavage of PARP protein, but also led to a substantial decrease of MCL1 level in both, Afatinib and Sorafenib combined treatments with CQ (Fig. 7c). These observations sustained the increased susceptibility of NB cells to RTKi when they are combined with CQ. Moreover, once again the results showed an initial activation of cyto-protective autophagy upon treatment of NB cells with RTKi that progressed toward apoptosis induction either upon use of cytotoxic doses of the RTKi, or upon combo treatment with CQ and sub-toxic doses of RTKi.

## Discussion

Receptor tyrosine kinases (RTKs) are important regulators of many cell processes including proliferation, growth, differentiation, survival and death induction [23]. Activation of RTKs is strictly regulated in the physiologic conditions, but this equilibrium can be lost in different pathologies, including cancer. In fact, RTKs are well known for their possible oncogenic potential that can be acquired once the regulation of their activity is impaired [24]. The most frequent causes of the constitutive activation of RTKs are mutations in their gene coding sequence, that ultimately lead to the conformational changes of the receptor and consequently to a persistent transduction of their signaling [25]. However, while this scenario is largely plausible for those cancers that develop in adults, the frequency of mutations in pediatric tumors is generally much lower, including those found in RTK-encoding genes [26, 27]. Neuroblastoma (NB) is a pediatric cancer that develops along the sympathetic nervous system [28]. Nowadays there are several RTKs whose deregulated activity has been reported along with more aggressive NB phenotypes [29–31]. Therefore, the introduction of RTK inhibitors (RTKi) in the therapy against different types of RTKs has been considered not only in adults, but also in pediatric tumors including NB. Indeed, many inhibitors have been tested or are currently under clinical trials for patients with NB [32, 33]. However, the use of RTKi in clinical practice revealed one very important problem: patients often develop resistance to RTKi [34]. This issue requires therapy modifications due to impaired drug efficacy, which are usually obtained by the introduction of another class of RTKi in order to achieve multi-RTK inhibition [9].

Autophagy is an evolutionary conserved process used by cells to provide energy. By recycling their own proteins and damaged organelles cells make use of autophagy to survive a prolonged stress condition. On the other side, the irregular function of autophagy has been connected with different types of disease including tumors [35]. Moreover, excessive autophagy activation has been observed during acquired resistance to diverse types of drugs among which also RTKi [36].

We previously tested two ALK inhibitors, namely Entrectinib and Crizotinib, revealing autophagy as an important process that contributed to the impaired drug toxicity in SH-SY5Y cells [17]. In the present study we included three additional RTKi (Afatinib, Sorafenib and TP-0903) that have been screened in our lab as potentially effective in killing NB tumor cells. Sorafenib is a multi-kinase inhibitor that effectively blocks multiple signaling pathways regulated by RAF, VEGF, PDGF, c-Kit and TGF-alpha receptors. Afatinib is an ATP-competitive compound that deactivates enzymatic action of ErbB family of cell membrane receptors (EGFR, HER2 and ErbB4), while TP-0903 is an inhibitor of AXL, Aurora A, ALK and MER activity [37]. Each of these RTKi has demonstrated a different level of efficacy when tested clinically or pre-clinically in various types of advanced cancers in adults. Afatinib and Sorafenib are currently under investigation for their clinical use in NB (ClinicalTrials.gov Identifier: NCT02372006) [38]. In order to answer whether these three RTKi could induce cyto-protective autophagy in NB cells that potentially could cause a resistance to these drugs, we performed mono- or combined-treatments with RTKi and autophagy blocking agents. We observed that Afatinib and Sorafenib resulted in autophagy activation, whereas TP-0903 slightly changed the level of basal autophagy already present in NB cells. Moreover, the combined treatments of RTKi and autophagy inhibitors were more effective, and they contributed to more pronounced cell death activation upon treatment with RTKi. These findings address that a combination of autophagy blocking agents and RTKi might prove to be useful therapeutic option for patients with NB. Importantly, a specific time frame might exist within which the impact of the selected autophagy inhibitors could be more pronounced, amplifying markedly the toxicity of RTKi. Our results also indicate that RTKi frequently activate autophagy in NB cells. Therefore, this biological process needs to be kept under surveillance for other RTKi, either new ones or those that may arise from the repurposing studies. Further pre-clinical evaluations will definitively answer whether the activity of RTKi can be improved by inhibiting autophagy. If this approach turns out to be more effective with respect to the use of RTKi alone, then we might increase the efficacy of RTKi, and prevail development of resistance phenomena in NB, both representing acute clinical problem at the moment.

## Conclusion

In the present report, we assessed the conditions under which autophagy is activated during the use of different RTKi currently in the pre-clinical evaluation for NB. We summarized the achievements of combined RTKi/autophagy inhibitors treatment as a promising approach to increase the efficacy of RTKi in impairing NB tumor cells viability.

## Additional files

**Additional file 1: Figure S1.** Caspase-3 and PI measurement upon treatments. A) Apoptosis activation was evaluated by measuring the intensity of PARP cleavage (89 kDa proteolytic PARP fragment), and by evaluating a decrease in the pro-Caspase-3 (35 kDa protein) level. Active Caspase-3 forms are observed as cleaved proteins with molecular weight below 20 kDa. GAPDH served as a loading control protein. D = DMSO; Af—Afatinib (8 μM); Sor—Sorafenib (14 μM); TP—TP-0903 (0.15 μM). B) Percentage of PI positive cells with respect to total cell number was presented as mean ± SD out of triplicates. D—DMSO; Af—Afatinib; Sor—Sorafenib; TP—TP-0903. p value is marked as **p < 0.01; n.s.—non-significant. C) Changes in the proliferation rate upon treatment with the three RTKi were determined by means of PCNA expression. The numbers indicate relative expression (densitometry) obtained after normalization to GAPDH protein level, which was used a loading control, and with respect to DMSO control (DMSO = 1).

**Additional file 2: Figure S2.** Colony formation assay. Effects on the capacity of SH-SY5Y cells to form colonies upon treatment with RTKi alone or in combined treatment with CQ or SP1 was measured. Upper image is the representative out of triplicate experiment. Lower graph represents a colony number calculated as a percentage of control (DMSO; 100%), and presented as the mean ± SD out of triplicate measurement. p value is marked as **p < 0.01; n.s.—non significant (Dunnett’s test). CQ—Chloroquine; SP1—Spautin-1; Af—Afatinib.

**Additional file 3: Figure S3.** Expression of extracellular signal-regulated kinase (ERK) upon treatments. Increasing concentrations of RTKi were used for the treatment, and levels of phospho-mTOR, phospho-PI3K, phospho-AKT and phospho-ERK (pERK) and total ERK were evaluated by western blot. GAPDH served as a loading control protein. D = DMSO; the numbers indicate the concentrations used in μM.

**Additional file 4: Figure S4.** Autophagy activation in SH-SY5Y cell line upon treatment with RTKi. Autophagy activation upon treatment with Afatinib and Sorafenib was validated using immunofluorescence in order to visualize a creation of autophagosomes; 60X magnification. DMSO treatment was used as control. Green—autophagy vacuoles; Blue—Hoechst, nuclear staining.

## Abbreviations

RTKs: receptor tyrosine kinases
RTKi: RTK inhibitors
CQ: Chloroquine
SP1: Spautin-1
AF: Afatinib
SOR: Sorafenib
TP: TP-0903
