# Supplementary figures and images for: Autophagy inhibition improves the cytotoxic effects of receptor tyrosine kinase inhibitors
Source: Cancer Cell Int. 2018 Apr 24;18:63. doi: 10.1186/s12935-018-0557-4 (PMC5916832; doi:10.1186/s12935-018-0557-4)

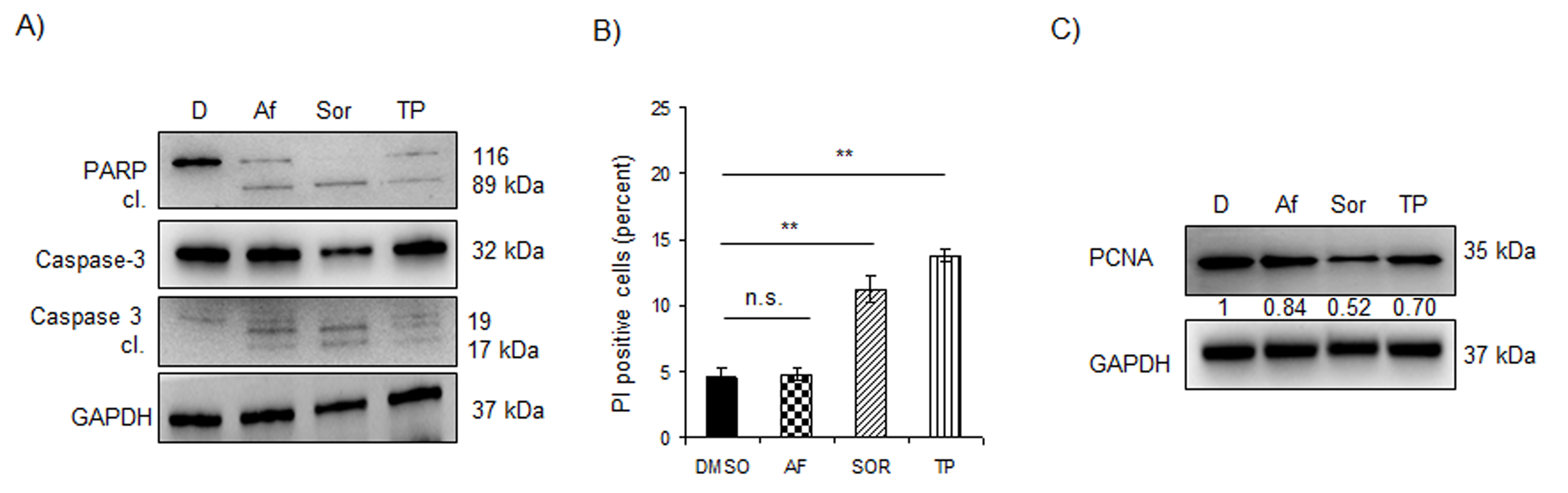

Supplement: Supplementary file 1 — Additional file 1: Figure S1. Caspase-3 and PI measurement upon treatments. A) Apoptosis activation was evaluated by measuring the intensity of PARP cleavage (89 kDa proteolytic PARP fragment), and by evaluating a decrease in the pro-Caspase-3 (35 kDa protein) level. Active Caspase-3 forms are observed as cleaved proteins with molecular weight below 20 kDa. GAPDH served as a loading control protein. D = DMSO; Af—Afatinib (8 μM); Sor—Sorafenib (14 μM); TP—TP-0903 (0.15 μM). B) Percentage of PI positive cells with respect to total cell number was presented as mean ± SD out of triplicates. D—DMSO; Af—Afatinib; Sor—Sorafenib; TP—TP-0903. p value is marked as **p < 0.01; n.s.—non-significant. C) Changes in the proliferation rate upon treatment with the three RTKi were determined by means of PCNA expression. The numbers indicate relative expression (densitometry) obtained after normalization to GAPDH protein level, which was used a loading control, and with respect to DMSO control (DMSO = 1). [file 12935_2018_557_MOESM1_ESM.tif]

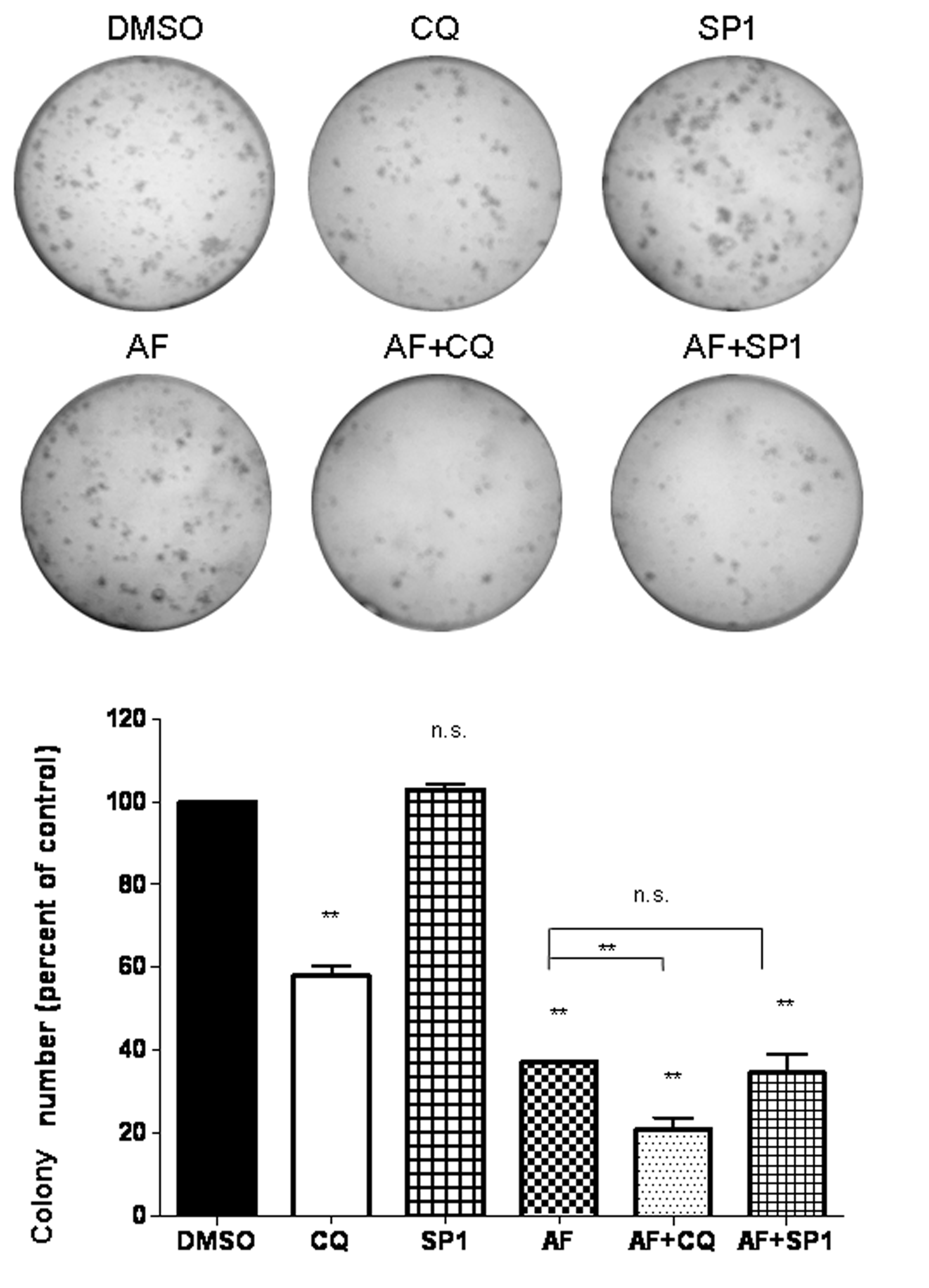

Supplement: Supplementary file 2 — Additional file 2: Figure S2. Colony formation assay. Effects on the capacity of SH-SY5Y cells to form colonies upon treatment with RTKi alone or in combined treatment with CQ or SP1 was measured. Upper image is the representative out of triplicate experiment. Lower graph represents a colony number calculated as a percentage of control (DMSO; 100%), and presented as the mean ± SD out of triplicate measurement. p value is marked as **p < 0.01; n.s.—non significant (Dunnett’s test). CQ—Chloroquine; SP1—Spautin-1; Af—Afatinib. [file 12935_2018_557_MOESM2_ESM.tif]

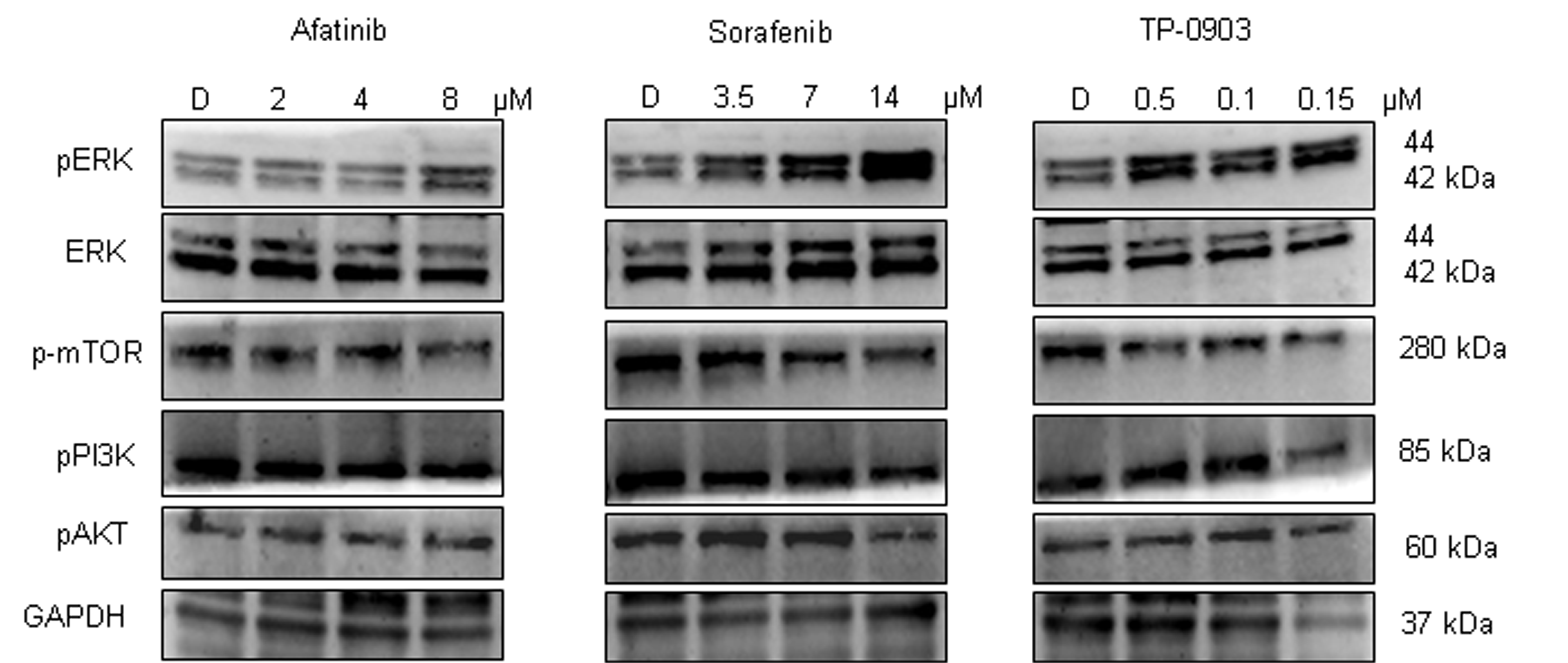

Supplement: Supplementary file 3 — Additional file 3: Figure S3. Expression of extracellular signal-regulated kinase (ERK) upon treatments. Increasing concentrations of RTKi were used for the treatment, and levels of phospho-mTOR, phospho-PI3K, phospho-AKT and phospho-ERK (pERK) and total ERK were evaluated by western blot. GAPDH served as a loading control protein. D = DMSO; the numbers indicate the concentrations used in μM. [file 12935_2018_557_MOESM3_ESM.tif]

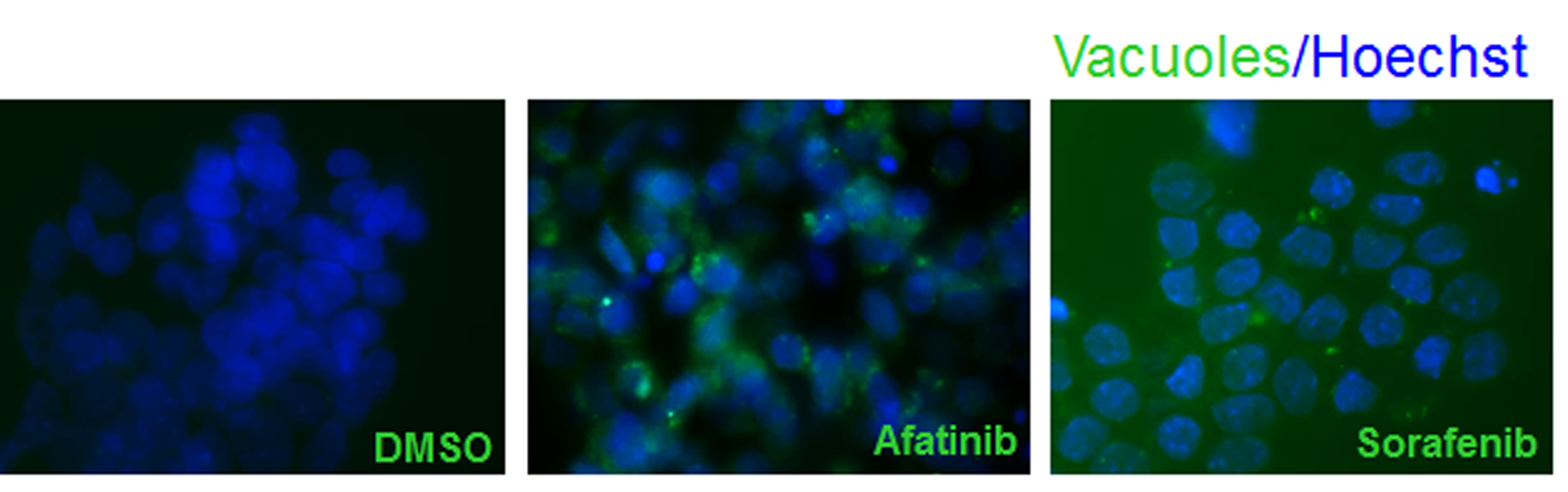

Supplement: Supplementary file 4 — Additional file 4: Figure S4. Autophagy activation in SH-SY5Y cell line upon treatment with RTKi. Autophagy activation upon treatment with Afatinib and Sorafenib was validated using immunofluorescence in order to visualize a creation of autophagosomes; 60X magnification. DMSO treatment was used as control. Green—autophagy vacuoles; Blue—Hoechst, nuclear staining. [file 12935_2018_557_MOESM4_ESM.tif]
